# Predictive Effect of Triglyceride Glucose−Related Parameters, Obesity Indices, and Lipid Ratios for Diabetes in a Chinese Population: A Prospective Cohort Study

**DOI:** 10.3389/fendo.2022.862919

**Published:** 2022-03-30

**Authors:** Xiaotong Li, Mengzi Sun, Yixue Yang, Nan Yao, Shoumeng Yan, Ling Wang, Wenyu Hu, Ruirui Guo, Yuxiang Wang, Bo Li

**Affiliations:** Department of Epidemiology and Biostatistics, School of Public Health, Jilin University, Changchun, China

**Keywords:** diabetes mellitus, triglyceride glucose index, obesity, lipid ratios, CHARLS

## Abstract

**Objective:**

The purpose of this study was to evaluate the association between triglyceride glucose (TyG) index and new-onset diabetes under different glycemic states and to compare the predictive value of TyG−related parameters, obesity indices, and lipid ratios for new-onset diabetes.

**Methods:**

Data were collected from the China Health and Retirement Longitudinal Study (CHARLS), consisting of 6,258 participants aged ≥45 years. Participants were grouped according to their glycemic states. Cox proportional hazards models and restricted cubic spline regression were used to explore the association between TyG index and diabetes. Cox proportional hazard models were applied to confirm the predictive value of the optimal marker. Receiver operating characteristic (ROC) curves were used to compare the predictive value.

**Results:**

TyG index was positively correlated with the risk of diabetes (hazard ratio (HR), 1.75; 95% confidence interval (CI), 1.56–1.97), and the linear association existed (*p* < 0.001). The highest correlation with diabetes was visceral adiposity index (VAI) (HR, 2.04; 95% CI, 1.44–2.90) in normal fasting glucose (NFG) group and TyG-body mass index (TyG-BMI) (HR, 2.53; 95% CI, 1.97–3.26) in impaired fasting glucose (IFG) group. The largest area under curve (AUC) was observed in TyG-waist-to-height ratio (TyG-WHtR) in the NFG group (AUC, 0.613; 95% CI, 0.527–0.700), and TyG-BMI had the highest AUC in the IFG group (AUC, 0.643; 95% CI, 0.601–0.685).

**Conclusion:**

The association between TyG index and new-onset diabetes was positive and linear. TyG-WHtR was a clinically effective marker for identifying the risks of diabetes in the NFG group and TyG-BMI was an effective marker to predict diabetes in the IFG group.

## Introduction

The increasing prevalence of diabetes has become a major public health problem worldwide, especially in developing countries (1). According to the latest diabetes map released by the International Diabetes Federation (IDF) in 2021, the number of people with diabetes worldwide will grow to 783 million by 2045, and China’s diabetes population has reached 140 million in 2021, ranking first in the world (2). Effective screening strategy is essential for identifying high-risk groups and reducing the incidence rate of diabetes.

The occurrence of diabetes can be predicted by relevant indicators (3–5). Insulin resistance (IR) plays an important role in the pathogenesis of diabetes and other metabolic-related diseases, which has already appeared before diabetes diagnosis (6, 7). Visceral obesity and ectopic fat deposition associated with IR lead to dyslipidemia and inflammation (8), which accelerated the development of diabetes. IR could be diagnosed by hyperinsulinemic-euglycemic clamp test (9) and homeostasis model assessment of IR (HOMA-IR) (10). However, it was inefficient for whole population screening due to the complex and expensive test process. Therefore, new markers or risk factors were needed to identify people at high risk of diabetes in order to implement prevention measures in the population.

In recent years, several studies have proposed new indicators for predicting diabetes, such as visceral adiposity index (VAI), a model based on anthropometry and laboratory parameters, and lipid accumulation product (LAP), based on the combination of TG and WC, which can be used to predict metabolic syndrome (11, 12). Triglyceride glucose (TyG) index and its related parameters were shown to be related to diabetes (13). However, the association between TyG index at different levels and diabetes was still inconsistent. A cohort study pointed out that there was a nonlinear relationship between the TyG index and incident T2DM (4). Studies have also indicated that total cholesterol (TC)/high-density lipoprotein cholesterol (HDL-C) and triglyceride (TG)/HDL-C can detect IR more effectively than simple lipid method (14). Some studies evaluated and compared the predictive value of TyG index, VAI, and LAP for new-onset diabetes (15–17), as well as the accuracy of the predictive value of various physical measurement indicators (3), but the conclusions of these studies were different for the best predictor of new-onset diabetes. No previous study specifically and comprehensively compared the accuracy of TyG-related parameters and these indicators in predicting the onset of diabetes, which should be verified in different ethnic groups. A study in China found that the incidence of diabetes in subjects with impaired fasting glucose (IFG) was more than six times higher than subjects with normal fasting glucose (NFG) (18). Therefore, the baseline blood glucose status of the population might also affect the accuracy of these indicators in predicting new-onset diabetes.

The aim of this study was to evaluate the correlation between TyG index and the risk of diabetes and to compare the predictive ability of TyG, TyG-body mass index (TyG-BMI), TyG-waist circumference (TyG-WC), TyG-waist-to-height ratio (TyG-WHtR), VAI, LAP, TG/HDL-C, and TC/HDL-C for the risk of new-onset diabetes under different glycemic states at follow-up in middle-aged and elderly Chinese population.

## Materials and Methods

### Study Population

**Figure 1:**
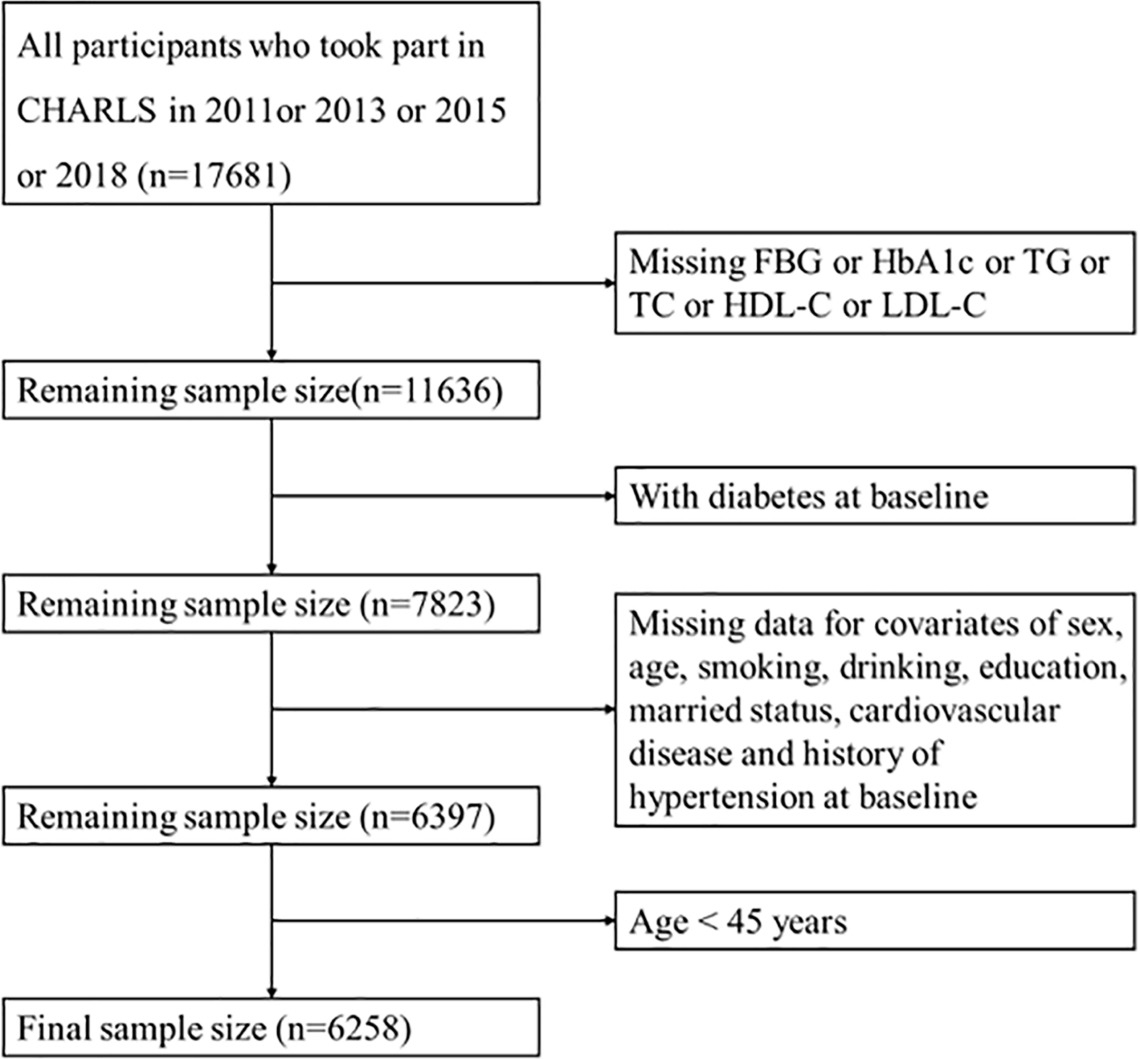
Flow chart of study participants.

The data used in this study were obtained from the China Health and Retirement Longitudinal Study (CHARLS), which was a longitudinal data of middle-aged and elderly people in China. The baseline wave of the study was conducted between June 2011 and March 2012, covered 28 provinces, 17,708 participants, with a response rate of 80.5%. Information on demographic, socioeconomic status, and health status of participants was collected using computer-assisted personal interview (CAPI) techniques. Follow-up surveys were conducted every 2 to 3 years, and so far, a second (2013), third (2015), and fourth (2018) wave have been conducted, of which blood samples were only collected from the baseline and third wave. The population that we included was no diabetes at baseline and followed up at least once. A total of 6,258 participants were included after removing the subjects with missing information on TG, TC, fasting blood glucose (FBG), hemoglobin A1c (HbA1c), HDL-C, and LDL-C at baseline or missing basic demographic characteristics and age <45 years (**Figure 1**).

All respondents were required to sign informed consent, and the ethical approval for data collection in CHARLS was approved by The Biomedical Ethics Review Committee of Peking University (IRB00001052-11015). The use of CHARLS data obtained ethical approval from the Human Research Ethics Committee of the University of Newcastle (H-2015-0290).

### Definition of Diabetes

Diabetes was defined as having FBG >125 mg/dl, HbA1c >6.5%, previous diagnosis of new-onset diabetes, or use of antidiabetic medications. IFG was defined as FBG of 100–125 mg/dl or HbA1c of 5.7%–6.4%. NFG was defined as without diabetes or prediabetes.

### Anthropometric Measurements and Serum Biochemical Parameters

Anthropometric measurements were performed by trained staff. Weight was quantified without shoes to the nearest 0.1 kg, vertical height meter was used to measure the height, and the measurement was accurate to 0.1 cm. Waist circumference was measured horizontally around the subject at the umbilical position and to the nearest 0.1 cm. Venous blood was collected on an empty stomach and transported by a cold-chain transport company to the Chinese Center for Disease Control and Prevention in Beijing. FBG, HbA1c, TG, TC, and HDL-C were measured by trained staff. The obesity- and TyG-related indices were calculated using the following formula:

(1) WHtR = WC/height.
With WC in centimeters, and height in centimeters (19).
(2) VAI (men) = [WC/39.68 + (1.88 × BMI)] × (TG/1.03) × (1.31/HDL-C).
VAI (women) = [WC/36.58 + (1.89 × BMI)] × (TG/0.81) × (1.52/HDL-C).
With WC in centimeters, BMI in kilograms per square meter, TG and HDL-C both in millimoles per liter (20).
(3) LAP (men) = [WC − 65] × TG.
LAP (women) = [WC − 58] × TG.
With WC in centimeters and TG in millimoles per liter (21).
(4) TyG = Ln [(TG × FBG)/2].
With TG and FBG both in milligrams per deciliter (22).
(5) TyG-BMI = TyG × BMI. TyG-WC = TyG × WC. TyG-WHtR = TyG × WHtR (23).

### Other Covariates

Through face-to-face questionnaire, the participants’ sex, age, smoking, drinking, educational level, marital status, history of hypertension, cardiovascular disease, and other information were obtained.

### Statistical Analysis

Data for quantitative variables were expressed as mean ± standard deviation (SD), and Student’s *t*-test was used for comparison between the two groups. Data for qualitative variables were expressed as numbers (percentage) and were compared using Pearson’s Chi-square test. Grouped by glycemic status at baseline, the cumulative incidence of each group was estimated by Kaplan–Meier method and compared by log-rank test. Participants were divided into four groups (Q1, Q2, Q3, Q4) based on the quartile of TyG, with quartile 1 as the reference group. Cox proportional hazards models were used to evaluate the association between the TyG index and new-onset diabetes. Model 2 was adjusted for age. Model 3 was adjusted for variables in model 2 plus drinking, education, hypertension, and cardiovascular disease. The dose–response association between the TyG index and the risk of diabetes was examined by restricted cubic spline model after adjustment for potential confounding factors. In order to compare the diagnostic value of different indicators for new-onset diabetes, four categories of TyG-BMI, TyG-WC, TyG-WHtR, VAI, LAP, TG/HDL-C, and TC/HDL-C were used as independent variables to calculate hazard ratio (HR) and 95% confidence interval (CI). The area under the receiver operating characteristic (ROC) curve (AUC) was used to test the predictive power of TyG, TyG-BMI, TyG-WC, TyG-WHtR, VAI, LAP, TG/HDL-C, and TC/HDL-C at baseline for the risk of emerging diabetes at follow-up. *p* < 0.05 was considered statistically significant. R Version 4.1.1 (R Foundation for Statistical Computing, Vienna, Austria) was used for all statistical analysis.

## Results

### Baseline Characteristics of Study Participants

**Figure 2:**
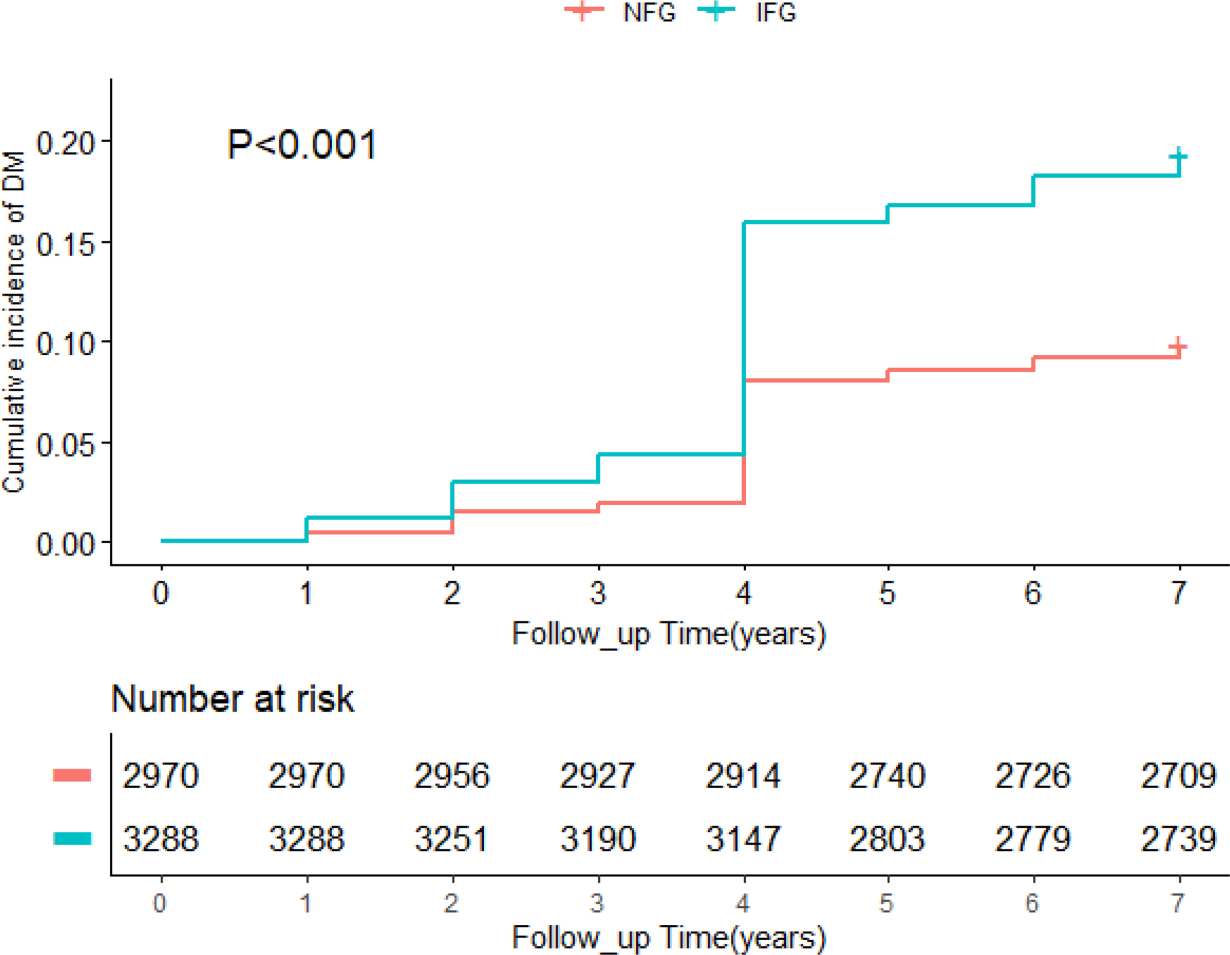
Kaplan–Meier analysis showing cumulative incidence of diabetes.

A total of 6,258 participants were included in the study, of whom 858 developed diabetes at follow-up. **Table 1** summarized the baseline characteristics of participants based on glycemic status and diabetes status at follow-up. The average age of the whole cohort was 58.51 years old and men accounted for 45.2%. In different glycemic status groups, compared with people without diabetes, participants with diabetes were older, had higher levels of TG, HDL-C, TyG, TyG-BMI, TyG-WC, TyG-WHtR, VAI, LAP, TG/HDL-C, and TC/HDL-C (*p* < 0.05), and were more likely to have hypertension (*p* < 0.001). In participants with normal blood glucose at baseline, no diabetes group had higher education level than the diabetes group (*p* = 0.022). In participants with impaired fasting glucose at baseline, the diabetes group was more likely to have cardiovascular disease (*p* = 0.003). During follow-up, the incidence of diabetes was 9.4% in the NFG and 17.6% in the IFG. As shown in **Figure 2**, Kaplan–Meier curves showed significant differences in the cumulative incidence of diabetes between baseline glucose states (log-rank test, *p* < 0.001), and those with higher glucose had a greater risk of diabetes over time.

**Table 1:** Demographic and clinical characteristics of the participants by glycemic status and diabetes status at follow-up.

	Total (*n* = 6,258)	NFG (*n* = 2,970)			IFG (*n* = 3,288)		
		Without diabetes (*n* = 2,691)	With diabetes (*n* = 279)	*p*-Value	Without diabetes (*n* = 2,709)	With diabetes (*n* = 579)	*p*-Value
Age	58.51 ± 8.80	57.75 ± 8.82	59.49 ± 9.14	0.002	58.89 ± 8.72	59.80 ± 8.59	0.022
Sex				0.597			0.122
Men	2,831 (45.2)	1,219 (45.3)	131 (47.0)		1,237 (45.7)	244 (42.1)	
Women	3,427 (54.8)	1,472 (54.7)	148 (53.0)		1,472 (54.3)	335 (57.9)	
Drinking				0.497			0.083
Yes	2,057 (32.9)	903 (33.6)	88 (31.5)		896 (33.1)	170 (29.4)	
No	4,201 (67.1)	1,788 (66.4)	191 (68.5)		1,813 (66.9)	409 (70.6)	
Smoking				0.772			0.811
Yes	2,381 (38.0)	1,056 (39.2)	107 (38.4)		1,001 (37.0)	217 (37.5)	
No	3,877 (62.0)	1,635 (60.8)	172 (61.6)		1,708 (63.0)	362 (62.5)	
Hypertension				<0.001			<0.001
Yes	1,349 (21.6)	491 (18.2)	77 (27.6)		588 (21.7)	193 (33.3)	
No	4,909 (78.4)	2,200 (81.8)	202 (72.4)		2,121 (78.3)	386 (66.7)	
Cardiovascular disease				0.303			0.003
Yes	662 (10.6)	257 (9.6)	32 (11.5)		287 (10.6)	86 (14.9)	
No	5,596 (89.4)	2,434 (90.4)	247 (88.5)		2,422 (89.4)	493 (85.1)	
Education				0.022			0.298
Primary school or lower	4,380 (70.0)	1,852 (68.8)	211 (75.6)		1,898 (70.0)	419 (72.4)	
Secondary school	1,807 (28.9)	808 (30.0)	68 (24.4)		775 (28.6)	156 (26.9)	
Higher	71 (1.1)	31 (1.2)	0 (0.0)		36 (1.3)	4 (0.7)	
Married status				0.415			0.118
Unmarried	31 (0.5)	14 (0.5)	0 (0.0)		13 (0.5)	4 (0.7)	
Married	5,342 (85.4)	2,306 (85.7)	237 (84.9)		2,322 (85.7)	477 (82.4)	
Widowed/divorced/separated	885 (14.1)	371 (13.8)	42 (15.1)		374 (13.8)	98 (16.9)	
BMI (kg/m^2^)	23.41 ± 3.83	22.95 ± 3.67	23.71 ± 4.35	0.005	23.55 ± 3.82	24.83 ± 3.91	<0.001
WC (cm)	83.71 ± 12.41	82.16 ± 12.23	84.86 ± 13.00	0.001	84.20 ± 12.25	88.07 ± 12.59	<0.001
WHtR	0.53 ± 0.08	0.52 ± 0.08	0.54 ± 0.09	0.001	0.53 ± 0.08	0.56 ± 0.08	<0.001
HDL-C (mmol/l)	1.35 ± 0.39	1.37 ± 0.37	1.32 ± 0.40	0.026	1.34 ± 0.40	v1.25 ± 0.40	<0.001
LDL-C (mmol/l)	3.04 ± 0.88	2.96 ± 0.80	2.98 ± 0.87	0.664	3.10 ± 0.94	3.18 ± 0.88	0.076
TC (mmol/l)	4.99 ± 0.97	4.84 ± 0.89	4.92 ± 0.96	0.160	5.10 ± 1.02	5.18 ± 0.94	0.076
FBG (mg/dl)	100.00 ± 11.65	90.89 ± 8.18	90.67 ± 10.46	0.732	107.78 ± 7.10	110.24 ± 7.36	<0.001
TG (mmol/l)	1.37 ± 0.85	1.19 ± 0.62	1.40 ± 0.82	<0.001	1.48 ± 0.96	1.67 ± 1.06	<0.001
TyG	8.56 ± 0.55	8.36 ± 0.49	8.50 ± 0.51	<0.001	8.70 ± 0.55	8.85 ± 0.55	<0.001
TyG-BMI	201.06 ± 38.59	192.29 ± 35.28	201.91 ± 40.03	<0.001	205.55 ± 38.93	220.42 ± 40.47	<0.001
TyG-WC	718.33 ± 126.22	688.06 ± 117.15	722.63 ± 125.29	<0.001	734.38 ± 126.13	781.46 ± 131.60	<0.001
TyG-WHtR	4.56 ± 0.82	4.36 ± 0.76	4.58 ± 0.83	<0.001	4.66 ± 0.82	4.96 ± 0.84	<0.001
VAI	94.92 ± 109.04	75.58 ± 70.44	94.99 ± 83.28	<0.001	105.14 ± 125.89	136.82 ± 155.98	<0.001
LAP	33.24 ± 33.39	26.62 ± 25.13	34.67 ± 32.49	<0.001	36.39 ± 36.79	48.49 ± 42.65	<0.001
TG/HDL-C	1.22 ± 1.23	1.00 ± 0.78	1.25 ± 1.08	<0.001	1.34 ± 1.43	1.63 ± 1.67	<0.001
TC/HDL-C	3.97 ± 1.32	3.74 ± 1.10	4.01 ± 1.33	<0.001	4.09 ± 1.38	4.49 ± 1.48	<0.001

NFG, normal fasting glucose; IFG, impaired fasting glucose; BMI, body mass index; WC, waist circumference; WHtR, waist-to-height ratio; HDL-C, high-density lipoprotein cholesterol; LDL-C, low-density lipoprotein cholesterol; TC, total cholesterol; FBG, fasting blood glucose; TG, triglyceride; TyG, triglyceride glucose; TyG-BMI, TyG related to BMI; TyG-WC, TyG related to WC; TyG-WHtR, TyG related to WHtR; VAI, visceral adiposity index; LAP, lipid accumulation product.

### Relation Between TyG Index and Incident Diabetes

**Figure 3:**
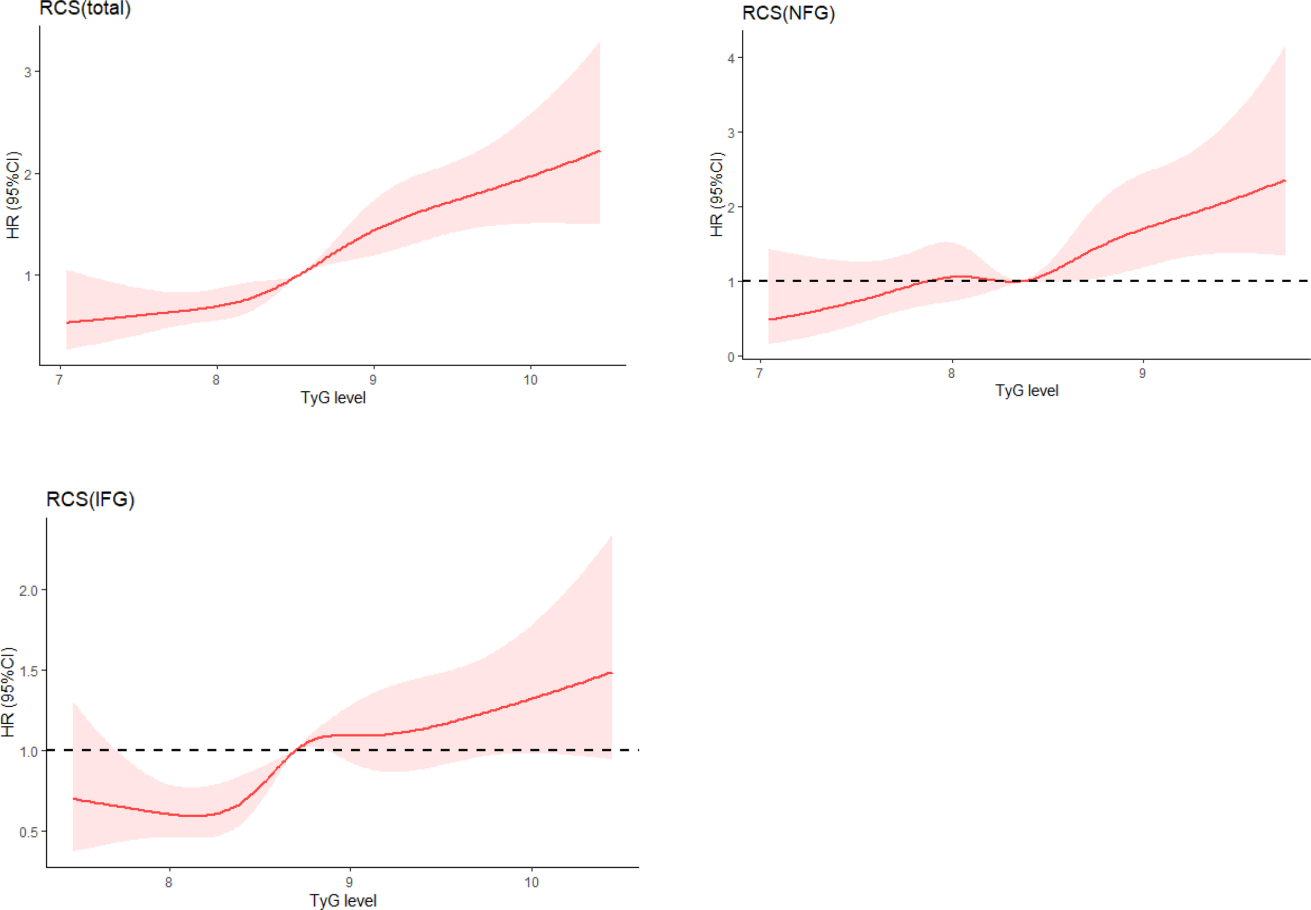
Adjusted cubic spline model of the association between triglyceride glucose index and risk of new-onset diabetes. NFG, normal fasting glucose; IFG, impaired fasting glucose; TyG, triglyceride glucose.

The univariate and multivariate analyses of TyG index with the incidence of diabetes are shown in **Table 2**. After adjusting for age, drinking, education, hypertension, and cardiovascular disease, TyG index was positively correlated with the risk of diabetes (HR, 1.75; 95% CI, 1.56–1.97) in the whole population, which was the same as the NFG and IFG groups. In order to verify the influence of different TyG levels on diabetes, we classified TyG index into quartiles. Compared with the lowest quartile, Q3 (HR, 1.93; 95% CI, 1.57–2.37) and Q4 (HR, 2.31; 95% CI, 1.89–2.82) had a significantly higher risk of developing diabetes. After adjusting for the potential confounding factors, the correlation still existed. The risk of diabetes in the highest quartile in the different glycemic status groups was 1.78 (95% CI: 1.28–2.49) and 2.00 (95% CI: 1.62–2.62). After adjusting for covariates, the statistical significance remained. In the restricted cubic spline regression model, the association between TyG index and the risk of diabetes was linear (nonlinear *p*-value >0.05) (**Table 2** and **Figure 3**).

**Table 2:** Cox proportional hazard models for the association between TyG index and incident diabetes.

	Incident diabetes						Nonlinear *p*-value
	Crude model 1		Model 2		Model 3		
	HR (95% CI)	*p*-Value	HR (95% CI)	*p*-Value	HR (95% CI)	*p*-Value	
Total							
TyG (continuous)	1.80 (1.61, 2.02)	<0.001	1.83 (1.63, 2.05)	<0.001	1.75 (1.56, 1.97)	<0.001	0.221
TyG (quartile)							
Q1	1		1		1		
Q2	1.16 (0.93, 1.46)	0.203	1.16 (0.92, 1.46)	0.202	1.13 (0.90, 1.42)	0.290	
Q3	1.93 (1.57, 2.37)	<0.001	1.93 (1.57, 2.37)	<0.001	1.82 (1.48, 2.24)	<0.001	
Q4	2.31 (1.89, 2.82)	<0.001	2.33 (1.91, 2.85)	<0.001	2.18 (1.78, 2.66)	<0.001	
NFG							
TyG (continuous)	1.74 (1.37, 2.20)	<0.001	1.75 (1.38, 2.21)	<0.001	1.69 (1.33, 2.15)	<0.001	0.665
TyG (quartile)							
Q1	1		1		1		
Q2	1.04 (0.72, 1.51)	0.826	1.03 (0.71, 1.50)	0.868	1.02 (0.71, 1.49)	0.889	
Q3	1.32 (0.93, 1.88)	0.117	1.32 (0.93, 1.87)	0.123	1.29 (0.91, 1.84)	0.150	
Q4	1.78 (1.28, 2.49)	<0.001	1.78 (1.28, 2.48)	<0.001	1.71 (1.22, 2.39)	0.002	
IFG							
TyG (continuous)	1.52 (1.32, 1.75)	<0.001	1.55 (1.34, 1.78)	<0.001	1.48 (1.28, 1.71)	<0.001	0.057
TyG (quartile)							
Q1	1		1		1		
Q2	1.26 (0.97, 1.64)	0.088	1.26 (0.96, 1.63)	0.091	1.21 (0.93, 1.58)	0.154	
Q3	1.69 (1.32, 2.17)	<0.001	1.70 (1.33, 2.18)	<0.001	1.60 (1.25, 2.05)	<0.001	
Q4	2.00 (1.62, 2.62)	<0.001	2.04 (1.60, 2.60)	<0.001	1.89 (1.48, 2.41)	<0.001	

HR, hazard ratio; CI, confidence interval; TyG, triglyceride glucose; NFG, normal fasting glucose; IFG, impaired fasting glucose.

Model 2 adjusted for age. Model 3 adjusted for age, drinking, education, hypertension, and cardiovascular disease.

### Associations of Indicators With Incident Diabetes

After stratification by glycemic status and adjusting for the influence of potential confounding factors, the results are shown in **Tables 3**, **4**. In the NFG group, compared with the lowest four percentiles, the Q4 of TyG-BMI, TyG-WC, TyG-WHtR, VAI, LAP, TG/HDL-C, and TC/HDL-C was correlated with the incidence of diabetes (*p* < 0.05), and VAI (HR, 2.04; 95% CI, 1.44–2.90) had the highest influence on the risk of diabetes. In the IFG group, compared with the lowest four percentiles, the Q3 and Q4 of TyG-BMI, TyG-WC, TyG-WHtR, VAI, LAP, TG/HDL-C, and TC/HDL-C were correlated with the incidence of diabetes(*p* < 0.05), and TyG-BMI (HR, 2.53; 95% CI, 1.97–3.26) had the highest influence on the risk of diabetes.

**Table 3:** Adjusted HR and 95% CI in quartiles of each index in the NFG group.

NFG (*N* = 2,970)	Incident diabetes					
	Crude model 1		Model 2		Model 3	
	HR (95% CI)	*p*-value	HR (95% CI)	*p*-value	HR (95% CI)	*p*-value
TyG-BMI						
Q1	1		1		1	
Q2	0.99 (0.69, 1.43)	0.971	1.05 (0.72, 1.51)	0.810	1.03 (0.71, 1.49)	0.882
Q3	1.14 (0.80, 1.63)	0.468	1.26 (0.88, 1.81)	0.204	1.23 (0.85, 1.76)	0.269
Q4	1.81 (1.31, 2.50)	<0.001	1.97 (1.42, 2.73)	<0.001	1.84 (1.31, 2.59)	<0.001
TyG-WC						
Q1	1		1		1	
Q2	0.86 (0.59, 1.25)	0.427	0.88 (0.60, 1.28)	0.491	0.87 (0.60, 1.27)	0.483
Q3	1.09 (0.77, 1.56)	0.623	1.12 (0.78, 1.59)	0.543	1.10 (0.77, 1.57)	0.600
Q4	1.88 (1.37, 2.59)	<0.001	1.91 (1.39, 2.62)	<0.001	1.82 (1.31, 2.52)	<0.001
TyG-WHtR						
Q1	1		1		1	
Q2	0.96 (0.66, 1.38)	0.796	0.96 (0.67, 1.39)	0.846	0.94 (0.65, 1.36)	0.758
Q3	1.16 (0.81, 1.65)	0.411	1.16 (0.82, 1.65)	0.405	1.11 (0.77, 1.58)	0.574
Q4	1.77 (1.28, 2.45)	<0.001	1.74 (1.26, 2.40)	<0.001	1.61 (1.15, 2.25)	0.006
VAI						
Q1	1		1		1	
Q2	1.21 (0.83, 1.76)	0.325	1.25 (0.86, 1.82)	0.241	1.25 (0.86, 1.82)	0.249
Q3	1.42 (0.98, 2.04)	0.061	1.49 (1.04, 2.15)	0.032	1.46 (1.01, 2.10)	0.046
Q4	2.05 (1.46, 2.88)	<0.001	2.15 (1.53, 3.03)	<0.001	2.04 (1.44, 2.90)	<0.001
LAP						
Q1	1		1		1	
Q2	0.69 (0.47, 1.01)	0.056	0.71 (0.48, 1.04)	0.075	0.70 (0.47, 1.02)	0.062
Q3	1.04 (0.74, 1.47)	0.809	1.07 (0.76, 1.51)	0.701	1.03 (0.73, 1.46)	0.857
Q4	1.65 (1.21, 2.26)	0.002	1.69 (1.23, 2.30)	0.001	1.57 (1.13, 2.17)	0.007
TG/HDL-C						
Q1	1		1		1	
Q2	1.06 (0.74, 1.53)	0.748	1.07 (0.74, 1.54)	0.728	1.07 (0.74, 1.54)	0.717
Q3	1.32 (0.93, 1.87)	0.125	1.33 (0.93, 1.88)	0.116	1.31 (0.92, 1.86)	0.138
Q4	1.71 (1.22, 2.38)	0.002	1.73 (1.24, 2.42)	0.001	1.67 (1.19, 2.33)	0.003
TC/HDL-C						
Q1	1		1		1	
Q2	0.99 (0.69, 1.42)	0.967	0.99 (0.69, 1.42)	0.946	0.98 (0.68, 1.40)	0.894
Q3	1.27 (0.90, 1.78)	0.169	1.29 (0.92, 1.81)	0.146	1.26 (0.90, 1.78)	0.180
Q4	1.46 (1.05, 2.03)	0.026	1.45 (1.05, 2.02)	0.026	1.41 (1.01, 1.96)	0.045

HR, hazard ratio; CI, confidence interval; NFG, normal fasting glucose; TyG-BMI, triglyceride glucose related to body mass index; TyG-WC, TyG related to waist circumference; TyG-WHtR, TyG related to waist-to-height ratio; VAI, visceral adiposity index; LAP, lipid accumulation product; TG/HDL-C, triglyceride to high-density lipoprotein cholesterol ratio; TC/HDL-C, total cholesterol to high-density lipoprotein cholesterol ratio.

Model 2 adjusted for age. Model 3 adjusted for age, drinking, education, hypertension, and cardiovascular disease.

**Table 4:** Adjusted HR and 95% CI in quartiles of each index in the IFG group.

IFG (*N* = 3,288)	Incident diabetes					
	Crude model 1		Model 2		Model 3	
	HR (95% CI)	*p*-value	HR (95% CI)	*p*-value	HR (95% CI)	*p*-value
TyG-BMI						
Q1	1		1		1	
Q2	1.28 (0.98, 1.67)	0.076	1.35 (1.03, 1.77)	0.030	1.33 (1.02, 1.75)	0.039
Q3	1.58 (1.22, 2.05)	<0.001	1.69 (1.30, 2.20)	<0.001	1.59 (1.22, 2.08)	<0.001
Q4	2.56 (2.01, 3.25)	<0.001	2.80 (2.19, 3.58)	<0.001	2.53 (1.97, 3.26)	<0.001
TyG-WC						
Q1	1		1		1	
Q2	1.31 (0.99, 1.72)	0.054	1.31 (0.99, 1.72)	0.054	1.29 (0.98, 1.70)	0.066
Q3	1.77 (1.36, 2.29)	<0.001	1.80 (1.39, 2.33)	<0.001	1.70 (1.31, 2.21)	<0.001
Q4	2.65 (2.08, 3.39)	<0.001	2.69 (2.10, 3.43)	<0.001	2.45 (1.91, 3.15)	<0.001
TyG-WHtR						
Q1	1		1		1	
Q2	1.29 (0.98, 1.70)	0.065	1.30 (0.99, 1.70)	0.058	1.28 (0.97, 1.68)	0.076
Q3	1.76 (1.37, 2.28)	<0.001	1.78 (1.38, 2.30)	<0.001	1.68 (1.30, 2.17)	<0.001
Q4	2.52 (1.98, 3.21)	<0.001	2.52 (1.97, 3.21)	<0.001	2.27 (1.77, 2.92)	<0.001
VAI						
Q1	1		1		1	
Q2	1.34 (1.03, 1.75)	0.028	1.36 (1.04, 1.77)	0.023	1.31 (1.01, 1.71)	0.045
Q3	1.70 (1.32, 2.18)	<0.001	1.74 (1.35, 2.23)	<0.001	1.62 (1.25, 2.09)	<0.001
Q4	2.13 (1.67, 2.71)	<0.001	2.22 (1.74, 2.84)	<0.001	2.01 (1.56, 2.58)	<0.001
LAP						
Q1	1		1		1	
Q2	1.08 (0.82, 1.42)	0.594	1.09 (0.83, 1.44)	0.523	1.05 (0.80, 1.38)	0.734
Q3	1.94 (1.52, 2.49)	<0.001	1.98 (1.55, 2.54)	<0.001	1.84 (1.44, 2.37)	<0.001
Q4	2.19 (1.72, 2.79)	<0.001	2.25 (1.77, 2.87)	<0.001	2.02 (1.57, 2.59)	<0.001
TG/HDL-C						
Q1	1		1		1	
Q2	1.19 (0.92, 1.55)	0.182	1.20 (0.93, 1.56)	0.162	1.17 (0.90, 1.52)	0.239
Q3	1.64 (1.29, 2.10)	<0.001	1.67 (1.30, 2.13)	<0.001	1.55 (1.22, 1.99)	<0.001
Q4	1.93 (1.52, 2.45)	<0.001	1.99 (1.57, 2.52)	<0.001	1.83 (1.44, 2.33)	<0.001
TC/HDL-C						
Q1	1		1		1	
Q2	1.16 (0.90, 1.51)	0.259	1.17 (0.90, 1.52)	0.234	1.13 (0.87, 1.47)	0.349
Q3	1.50 (1.17, 1.92)	<0.001	1.52 (1.19, 1.94)	<0.001	1.43 (1.11, 1.83)	0.004
Q4	1.97 (1.56, 2.49)	<0.001	2.00 (1.58, 2.53)	<0.001	1.86 (1.46, 2.35)	<0.001

HR, hazard ratio; CI, confidence interval; IFG, impaired fasting glucose; TyG-BMI, triglyceride glucose related to body mass index; TyG-WC, TyG related to waist circumference; TyG-WHtR, TyG related to waist-to-height ratio; VAI, visceral adiposity index; LAP, lipid accumulation product; TG/HDL-C, triglyceride to high-density lipoprotein cholesterol ratio; TC/HDL-C, total cholesterol to high-density lipoprotein cholesterol ratio.

Model 2 adjusted for age. Model 3 adjusted for age, drinking, education, hypertension, and cardiovascular disease.

### The Predictive Value of Each Index for Diabetes

**Figure 4:**
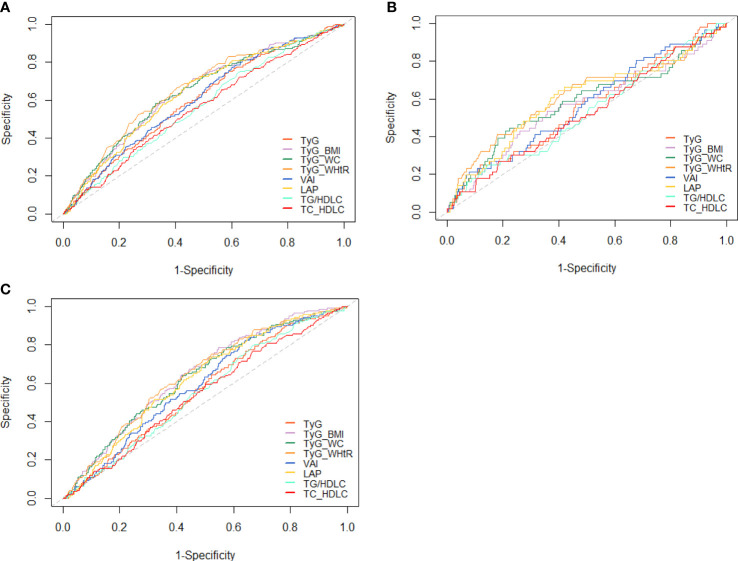
ROC curves for each index as predictors of diabetes. **(A)** Whole cohort, **(B)** NFG, and **(C)** IFG. ROC, receiver-operating characteristic; TyG, triglyceride glucose; TyG-BMI, TyG related to body mass index; TyG-WC, TyG related to waist circumference; TyG-WHtR, TyG related to waist-to-height ratio; VAI, visceral adiposity index; LAP, lipid accumulation product; TG/HDL-C, triglyceride to high-density lipoprotein cholesterol ratio; TC/HDL-C, total cholesterol to high-density lipoprotein cholesterol ratio.

ROC curves for different indices are presented in **Figure 4**. The cutoff value and AUC with sensitivity, specificity, and Youden index are presented in **Table 5**. In the whole study population, TyG-WHtR had the highest AUC (AUC, 0.658; 95% CI, 0.619–0.696), followed by TyG-BMI (AUC, 0.644; 95% CI, 0.605–0.682) and TyG-WC (AUC, 0.642; 95% CI, 0.603–0.682). The optimal cutoff of TyG-WHtR, TyG-BMI, and TyG-WC were 4.99, 209.89, and 764.61. After stratifying based on the level of blood glucose, TyG-WHtR (AUC, 0.613; 95% CI, 0.527–0.700) had the highest diagnostic value in the NFG group, followed by LAP (AUC, 0.601; 95% CI, 0.528–0.684) and TyG-WC (AUC, 0.585; 95% CI, 0.499–0.671). However, in the IFG group, TyG-BMI (AUC, 0.643; 95% CI, 0.601–0.685) had the highest diagnostic value for diabetes, followed by TyG-WHtR (AUC, 0.639; 95% CI, 0.596–0.682) and TyG-WC (AUC, 0.630; 95% CI, 0.586–0.674).

**Table 5:** Sensitivity, specificity, Youden index, cutoff points, and AUC (95% CI) for each index in predicting diabetes risk among adults in China.

	Cut-off	AUC (95% CI)	Sensitivity (%)	Specificity (%)	Youden index
Total					
TyG	8.41	0.597 (0.559, 0.636)	74.11	41.91	16.02
TyG-BMI	209.89	0.644 (0.605, 0.682)	58.88	64.43	23.31
TyG-WC	764.61	0.642 (0.603, 0.682)	57.87	67.15	25.02
TyG-WHtR	4.99	0.658 (0.619, 0.696)	52.28	72.86	25.14
VAI	77.02	0.603 (0.565, 0.641)	52.28	59.17	11.45
LAP	32.75	0.635 (0.597, 0.674)	59.39	62.61	22.00
TG/HDL-C	0.98	0.565 (0.526, 0.604)	50.25	56.76	7.01
TC/HDL-C	3.95	0.557 (0.517, 0.597)	52.28	57.22	9.50
NFG					
TyG	8.47	0.558 (0.481, 0.635)	46.43	59.33	5.76
TyG-BMI	211.42	0.565 (0.481, 0.648)	42.86	73.99	16.85
TyG-WC	783.71	0.585 (0.499, 0.671)	39.29	81.13	20.42
TyG-WHtR	4.54	0.613 (0.527, 0.700)	60.71	60.60	21.31
VAI	77.79	0.568 (0.491, 0.644)	42.86	66.30	9.16
LAP	31.13	0.601 (0.528, 0.684)	51.79	67.91	19.70
TG/HDL-C	0.69	0.532 (0.454, 0.610)	62.50	41.63	4.13
TC/HDL-C	3.45	0.526 (0.448, 0.605)	55.36	45.68	1.04
IFG					
TyG	8.56	0.562 (0.517, 0.606)	69.50	40.86	10.36
TyG-BMI	222.56	0.643 (0.601, 0.685)	51.06	68.26	19.32
TyG-WC	764.61	0.630 (0.586, 0.674)	63.12	58.31	21.43
TyG-WHtR	4.99	0.639 (0.596, 0.682)	56.74	65.14	21.88
VAI	67.49	0.590 (0.547, 0.633)	65.25	47.89	13.14
LAP	29.36	0.619 (0.576, 0.662)	69.50	51.00	20.50
TG/HDL-C	0.99	0.552 (0.507, 0.596)	56.03	51.35	7.38
TC/HDL-C	3.89	0.544 (0.497, 0.590)	59.57	49.19	8.76

AUC, area under curve; CI, confidence interval; NFG, normal fasting glucose; IFG, impaired fasting glucose; TyG, triglyceride glucose; TyG-BMI, TyG related to body mass index; TyG-WC, TyG related to waist circumference; TyG-WHtR, TyG related to waist-to-height ratio; VAI, visceral adiposity index; LAP, lipid accumulation product; TG/HDL-C, triglyceride to high-density lipoprotein cholesterol ratio; TC/HDL-C, total cholesterol to high-density lipoprotein cholesterol ratio.

### Subgroup Analyses

**Table 6** shows the results stratified by age. The predictive value of TyG-WHtR for new-onset diabetes was highest among participants aged <65 years. The predictive value of all indicators for new-onset diabetes was generally low in the NFG participants aged ≥65 years. TyG-BMI had the highest predictive value for new-onset diabetes among participants aged ≥65 years in the IFG group.

**Table 6:** Sensitivity, specificity, and AUC (95%CI) for each index in predicting diabetes risk by age.

	NFG			IFG		
	AUC (95% CI)	Sensitivity (%)	Specificity (%)	AUC (95% CI)	Sensitivity (%)	Specificity (%)
Age <65						
TyG	0.598 (0.511, 0.686)	30.95	87.73	0.541 (0.493, 0.589)	82.20	29.18
TyG-BMI	0.605 (0.513, 0.687)	59.52	64.45	0.623 (0.574, 0.671)	77.97	41.28
TyG-WC	0.627 (0.531, 0.722)	47.62	77.77	0.618 (0.568, 0.668)	44.92	74.07
TyG-WHtR	0.671 (0.578, 0.765)	73.81	58.8	0.628 (0.580, 0.676)	58.47	62.58
VAI	0.590 (0.505, 0.675)	76.19	39.10	0.560 (0.513, 0.608)	83.90	31.51
LAP	0.644 (0.552, 0.735)	69.05	61.50	0.601 (0.554, 0.649)	72.88	46.57
TG/HDL-C	0.539 (0.447, 0.631)	28.57	85.45	0.525 (0.477, 0.574)	78.81	31.92
TC/HDL-C	0.524 (0.434, 0.615)	19.05	89.61	0.517 (0.467, 0.568)	74.58	32.05
Age ≥65						
TyG	0.438 (0.292, 0.584)	7.14	99.83	0.641 (0.528, 0.754)	69.57	60.00
TyG-BMI	0.458 (0.285, 0.630)	42.86	69.18	0.710 (0.619, 0.801)	82.61	61.69
TyG-WC	0.465 (0.291, 0.639)	35.71	79.28	0.680 (0.588, 0.771)	91.30	47.75
TyG-WHtR	0.441 (0.263, 0.619)	21.43	87.50	0.695 (0.599, 0.792)	65.22	70.00
VAI	0.511 (0.350, 0.672)	42.86	72.43	0.705 (0.606, 0.804)	82.61	53.52
LAP	0.478 (0.313, 0.643)	35.71	75.86	0.684 (0.585, 0.783)	60.87	74.37
TG/HDL-C	0.519 (0.378, 0.660)	92.86	21.75	0.660 (0.552, 0.767)	69.57	60.28
TC/HDL-C	0.529 (0.374, 0.685)	35.71	80.31	0.658 (0.547, 0.769)	56.52	74.23

NFG, normal fasting glucose; IFG, impaired fasting glucose; AUC, area under curve; CI, confidence interval; TyG, triglyceride glucose; TyG-BMI, TyG related to body mass index; TyG-WC, TyG related to waist circumference; TyG-WHtR, TyG related to waist-to-height ratio; VAI, visceral adiposity index; LAP, lipid accumulation product; TG/HDL-C, triglyceride to high-density lipoprotein cholesterol ratio; TC/HDL-C, total cholesterol to high-density lipoprotein cholesterol ratio.

## Discussion

In this cohort study, we explored the association between TyG index and new-onset diabetes in different glycemic status and directly compared the predictive value of TyG, TyG-BMI, TyG-WC, TyG-WHtR, VAI, LAP, TG/HDL-C, and TC/HDL-C indicators in new-onset diabetes. Overall, we found that a positive correlation between TyG index and the risk of diabetes existed, and the relationship was linear. In addition, all indices exhibited the capability to identify individuals with diabetes (AUC >0.5 for all), and TyG-WHtR was superior to other indicators for predicting diabetes in the whole subjects and NFG subgroup. However, in the IFG subgroup, TyG-BMI had higher predictive ability for diabetes than other indicators.

The increase of glucose concentration can elevate the level of reactive oxygen species, and then produce toxic effects on β cells (24). The increase of TG level in blood was negatively correlated with insulin secretion (25) and would lead to ectopic fat deposition in the body and the increase of triglyceride level in muscle cells, resulting in IR (26). Also, excessive TG in pancreatic islet cells can disrupt β-cell function (27). TyG index incorporated the compound effect of both, which was a simple index to detect IR. Previous studies indicated that the risk of diabetes was elevated with the increase of TyG level (divided into four quantiles) (13). However, some studies showed that there was a nonlinear association between them (4, 28). The association between TyG index and the risk of diabetes in our study was positively and linear. The reason was that the subjects of our study were middle aged and elderly, whose TyG index was generally high. This was also consistent with previous studies suggesting that high TyG index was relevant to future risk of diabetes in different races (29, 30). The results of the IFG group were similar to the whole subjects. However, in the NFG group, the correlation between TyG index and diabetes only existed when the TyG index was high. Compared with group NFG, impaired fasting glucose was more likely to be related with IR, which explained the higher correlation between TyG index and new-onset diabetes in IFG group (31). In this context, the TyG index could be considered a potential and reliable prognosticator for the incidence of diabetes for broad clinical usage.

However, some studies showed that obesity and lipid indicators were also good predictors of new-onset diabetes (3, 5, 17). The association between obesity and diabetes was mentioned in several studies (32, 33). Compared with general obesity and subcutaneous fat, visceral fat accumulation had a significant negative effect on blood glucose control by reducing peripheral insulin sensitivity and enhancing gluconeogenesis, which was closely related to IR (34). Visceral fat accumulation might also induce the secretion of adipocytokines. Oversecretion of proinflammatory adipocytokines and hyposecretion of defensive adipocytokines might be the main mechanism of IR and T2DM (35). Some simple anthropometric parameters were used as surrogate indicators of visceral fat, such as WC and WHtR, but these classic indicators could not take metabolic measures into account.

Our study indicated that the predictive value of TyG-related parameters combined with anthropometric parameters was superior to TyG index in new-onset diabetes. TyG-related parameters were useful clinical substitutes for predicting new-onset diabetes. Because they combined TG, FBG, and obesity indicators, the role of which in identifying IR was validated in previous studies (5). The utility of TyG index in evaluating IR was pointed out in a number of studies (36, 37). However, there were still controversy about the predictive value of TyG index and TyG-related parameters. A study in Chinese elderly population found that TyG index had higher predictive ability than TyG-related parameters (23). However, TyG-BMI and TyG-WC were significantly better than TyG index in predicting the risk of T2DM in Korean population (5), which was consistent with our conclusion.

Another important result of our study was that TyG-WHtR was superior to other TyG-related parameters in identifying the risk of early diabetes in the NFG group. A cohort study in Iran showed that WHtR was a better predictor than BMI and WC (38). A systematic review and meta-analysis (39) also showed a stronger association between WHtR and T2DM than BMI. This maybe because WHtR reflected the effect of visceral fat better than WC. Because metabolic risk was different in people with the same WC but different heights, and height was usually inversely associated with cardiometabolic morbidity and mortality (40). IFG refers to liver IR and early insulin secretion defects, along with impaired β-cell function (41, 42). Early identification of high-risk groups of diabetes is crucial in the occurrence of IFG. Our data showed that in the IFG group, TyG-BMI had the highest predictive value for new-onset diabetes. In our study, TyG-BMI predicted that new-onset diabetes was more effective than TyG-WC and TyG-WHtR in IFG group, probably because the population had high levels of systemic obesity. Abdominal fat includes subcutaneous fat and visceral fat, and visceral fat plays an important role in the pathogenesis of IR. However, WC cannot separate subcutaneous adipose tissue from visceral adipose tissue, so abdominal obesity cannot be accurately measured (5), leading to inaccurate measurement results of WHtR. In another study, the intraobserver and interobserver variability of waist circumference was higher than that of body mass index (43), and the accuracy of WC measurement was affected by its measurement location (44).

VAI and LAP were comprehensive measures that combined lipid variables with obesity status and were predictors of diabetes mellitus (45). Our study found that in the whole population and IFG group, the predictive value of VAI and LAP for diabetes was weaker than that of TyG-related parameters but higher than that of lipids. However, it was worth noting that the predictive value of LAP for diabetes was only next to TyG-WHtR in NFG group, and VAI had the most significant correlation with new-onset diabetes. The possible explanation was that the NFG population had better glycemic regulation than IFG population, so the effect of glycotoxicity on NFG people was slight. Therefore, VAI, which represented obesity status and lipid level, was closely associated with diabetes in NFG population. Furthermore, our study found that although there was a strong association between lipid ratio and new-onset diabetes, the predictive ability of both to new-onset diabetes was lower than other indicators. There was evidence showing that lipid ratios, such as TG/HDL-C and TC/HDL-C, were more effective than single lipid measurements in detecting IR (14). Also, a cohort study in China demonstrated that TyG, VAI, and LAP were mostly superior than TG/HDL-C in predicting T2DM (15).

The main strength of our research is that we are the first to analyze the predictive value of TyG-related parameters, visceral obesity index, and lipid ratio for new-onset diabetes under different glycemic states. The conclusion of this study has an important guiding role for clinicians to identify high-risk groups and predict the occurrence of diabetes in the future. Moreover, this is a prospective study with long-term follow-up in middle-aged and elderly Chinese population. Several limitations may exist in this study. First of all, the study population is only composed of middle-aged and elderly people. It is necessary to be cautious to extend the research results to other populations. Secondly, we did not use the 2-h oral glucose tolerance test to detect cases of diabetes, so the incidence might have been underestimated. Finally, we could not evaluate the HOMA-IR in our study.

## Conclusion

The association between TyG index and new-onset diabetes was positive and linear. For predicting diabetes, TyG-WHtR was a valuable marker for predicting the risk of new-onset diabetes in the NFG group and the whole population. The predictive value of TyG-BMI was higher in the NFG group. We suggest that this index should be used in clinical practice or epidemiological investigation for early detection of diabetes.

## Data Availability Statement

Publicly available datasets were analyzed in this study. These data can be found here: http://charls.pku.edu.cn/.

## Ethics Statement

All respondents were required to sign informed consent, and the ethical approval for data collection in CHARLS was approved by The Biomedical Ethics Review Committee of Peking University (IRB00001052-11015). The use of CHARLS data obtained ethical approval from the Human Research Ethics Committee of the University of Newcastle (H-2015-0290).

## Author Contributions

XL, BL and MS made the study design. MS, SY, and YW conducted the study. XL, RG, and NY analyzed the data and wrote the manuscript. LW, WH, and YY attended the manuscript revision. All authors agreed with the final manuscript.

## Funding

This work was supported by the National Natural Science Foundation of China (No. 81973129).

## Conflict of Interest

The authors declare that the research was conducted in the absence of any commercial or financial relationships that could be construed as a potential conflict of interest.

## Publisher’s Note

All claims expressed in this article are solely those of the authors and do not necessarily represent those of their affiliated organizations, or those of the publisher, the editors and the reviewers. Any product that may be evaluated in this article, or claim that may be made by its manufacturer, is not guaranteed or endorsed by the publisher.
